# Identification of a novel *RPGR* mutation associated with X-linked cone-rod dystrophy in a Chinese family

**DOI:** 10.1186/s12886-021-02166-0

**Published:** 2021-11-20

**Authors:** Yafang Wang, Shu Liu, Yuanqi Zhai, Yang Liu, Xiaoling Wan, Wenqiu Wang, Fenghua Wang, Xiaodong Sun

**Affiliations:** 1 Department of Ophthalmology, Shanghai General Hospital (Shanghai First People’s Hospital), Shanghai Jiao Tong University School of Medicine, 100 Haining Road, Shanghai, 200080 China; 2 Shanghai Key Laboratory of Ocular Fundus Diseases, 100 Haining Road, Shanghai, 200080 China; 3 Shanghai Engineering Center for Visual Science and Photomedicine, 100 Haining Road, Shanghai, 200080 China; 4 National Clinical Research Center for Eye Diseases, 100 Haining Road, Shanghai, 200080 China; 5 Shanghai Engineering Center for Precise Diagnosis and Treatment of Eye Diseases, 100 Haining Road, Shanghai, 200080 China

**Keywords:** Cone-rod dystrophy, *RPGR*, Mutation

## Abstract

**Background:**

Cone-rod dystrophy (CORD) is a group of inherited retinal dystrophies, characterized by decreased visual acuity, color vision defects, photophobia, and decreased sensitivity in the central visual field. Our study has identified a novel pathogenic variant associated with X-linked cone-rod dystrophy (XLCORD) in a Chinese family.

**Methods:**

All six family members, including the proband, affected siblings, cousins and female carriers, have underwent thorough ophthalmic examinations. The whole exome sequencing was performed for the proband, followed by Sanger sequencing for spilt-sample validation. A mammalian expression vector (AAV-MCS) with mutated retinitis pigmentosa GTPase regulator (*RPGR*) sequence was expressed in HEK293 T cells. The mutated protein was verified by Western blotting and immunohistochemistry.

**Results:**

A novel mutation in the *RPGR* gene (c.2383G > T, p.E795X) is identified to be responsible for CORD pathogenesis.

**Conclusions:**

Our findings have expanded the spectrum of CORD-associated mutations in *RPGR* gene and serve as a basis for genetic diagnosis for X-linked CORD.

**Supplementary Information:**

The online version contains supplementary material available at 10.1186/s12886-021-02166-0.

## Background

Inherited retinal dystrophies (IRDs) are a highly heterogeneous group of disorders characterized by progressive dysfunction of photoreceptors and retinal pigment epithelium (RPE) cells. Cone-rod dystrophy (CORD) is one of the subgroup of IRDs, characterized by the primary degeneration of cone photoreceptors.

CORD presents with decreased visual acuity, color vision defects, photophobia and decreased sensitivity in the central visual field, followed by progressive loss in peripheral visual field [1]. Its estimated prevalence is 1/40000 [2]. Typical fundus imaging of CORD patients presents pigmentary deposits resembling bone spicules (frequently in macular area), retinal vessels attenuation, pallid optic disc and various degrees of retinal atrophy [3]. The electroretinogram (ERG) shows a shift in implicit time of cone responses, followed by a decrease in both cone and rod responses, and cone responses are more severely affected than rod responses [3].

Currently, mutations in 22 genes and loci have been reported to be associated with CORD [4]. The *RPGR* gene is a major cause of X-linked CORD cases [4]. Multiple isoforms of *RPGR* have been detected in the retina with *RPGR*^1–19^, which spans 19 exons and encodes an 815-aa polypeptide, and *RPGR*^ORF15^, which spans 15 exons plus a part of intron 15 and encodes a 1152-aa polypeptide [5–7] as the two major isoforms. *RPGR*^1–19^ is widely distributed in ciliated tissues, whereas *RPGR*^ORF15^ is found primarily in the connecting cilia of photoreceptor cells, especially in the outer segment of rod photoreceptors [8]. Due to the presence of highly repetitive purine-rich sequences, the exon ORF15 (exon ORF15 + part of intron 15) of *RPGR* is prone to mutagenesis accounting for most XLCORD cases.

In this study, we reported a Chinese family with four male CORD patients carrying a novel nonsense mutation in the exon ORF15 (c.2383G > T, p.E795X) of *RPGR* gene, which broaden the spectrum of *RPGR* mutations associated with X-linked CORD.

## Methods

### Patients and clinical assessment

The study was adhered to the tenets of the Declaration of Helsinki and approved by the medical ethics committee of Shanghai General Hospital (Shanghai First People’s Hospital). All the members were informed the research and consented to it. And they were all enquired about the family and medical history before taking ophthalmological examinations. Then each member underwent detailed ophthalmic examinations, including best-corrected visual acuity (BCVA)[RT-5100,NIDEX], intraocular pressure (IOP)[TX-20,Canon], optical coherence tomography (OCT) scans [SpectralisOCT, Heidelberg], widefield color fundus imaging and widefield fundus autofluorescence (FAF) [200TX, OPTOS]. In addition, visual field [Carl Zeiss Meditec] and full-field electroretinogram (ERG) [RETI-Port/scan 21] were performed to estimate visual function.

### Whole-exome sequencing (WES)

The genomic DNA extracted from peripheral blood samples of the members were collected for whole-exome sequencing (WES). Illumina paired-end libraries were generated according to the Kapa LTP library prep kit protocol (Roche, Basel, Switzerland). Agilent SSELXT Human All Exon V6 was used for whole exome sequencing. The enriched DNA library was sequenced on Illumina Xten Analyzers for 150 cycles per read to generate paired-end reads (following the manufacturer’s standard sequencing protocols). Raw reads were aligned to the human genome reference (hg19) using the BWA (Burrows Wheeler Aligner). Single-nucleotide variants (SNVs) and InDels (Insertions and Deletions) were called by Atlas-SNP2 and Atlas-Indel, respectively. The frequency of all SNVs and InDels were annotated using the ExAC, gnomAD, HGVD, CHARGE, 1000 Genome, UK10K databases and the internal database of Clinbytes Inc. to filter the common variants, with a allele frequency cutoff of 0.5 and 0.1% for recessive and dominant variants, respectively. The WES analysis was provided by Clinbytes.

### Assessment of the pathogenicity of candidate variants

PCR amplification and Sanger sequencing were used to further validate candidate variations. Each genomic sequence was obtained from the UCSC genome browser (hg19). The repetitive sequences was masked using RepeatMasker in the huamn genome (available at http://www.repeatmasker.org/cgi-bin/WEBRepeatMasker/). Specific forward and reverse primers were designed at least 100 bp from the variants to amplify 300–500 bp fragments. The amplicons were analyzed by Sanger sequencing.

### Prediction of protein structure

The amino acid sequences of both wild type and mutant *RPGR*^ORF15^ have been processed with the I-TASSER web server. Models were visualized and then exported by PyMol (The PyMOL Molecular Graphics System, Version 1.8 Schrödinger, LLC).

### Cloning and plasmid construction

The wild type RPGR^ORF15^(RefSeq NM_001034853.2) was amplified by PCR with primers 5′-agagagcccgaggagctg-3′ and 5′-atgactcgagtcacttcagctccaggtag-3′. The c.2383G > T nonsense mutation was introduced by PCR with primers 5′- agagagcccgaggagctg-3′ and 5′-atgactcgagtcagccctgatcgccttcctc-3′. The plasmid which an HA tag was added in constructs (AAV-MCS) were verified by sanger sequencing. 293 T cells were employed to test the expression of RPGR expressing constructs.

### Cell culture and transfection

293 T cells were obtained from the American Type Culture 141 Collection (CRL-2302, Manassas, VA, USA) and cultured in DMEM/ High glucose with 10% FBS. The above plasmids’ transfection was executed by Lipofectamine 3000 (Invitrogen, Carlsbad, CA, USA) according to the manufacturer’s protocol. Samples would be harvested 48 h later [9].

### Western blot

Transfected Cells were lysed with RIPA buffer containing protease and phosphatase inhibitors. Samples (10 μg total protein) were seperated by 10% SDS-PAGE gels and transferred to electroblotted to polyvinylidene difluoride (PVDF) membranes (IPVH00011, Solarbio). The membranes were blocked by 5% nonfat dry milk in Tris-buffered saline (TBS), containing Tween-20 (TBST) for 1 h at room temperature then incubated with primary antibodies against HA (AF0039 Beyotime) at 4 °C overnight. The membranes were washed with TBST three times for 10 min then incubated with the corresponding secondary antibodies (Rabbit, Proteintech) at room temperature for 1 h. After washing with TBST three times for 10 min, the membranes were visualized by the molecular imaging system (Amersham Imager 600, GE Healthcare, Buckinghamshire, UK).

### Immunofluorescence

Cells were fixed with 4% paraformaldehyde for 30 min and blocked with PBS containing 5% goat serum albumin (Beyotime) and 0.05% Triton for 1 h. Then, the cells were incubated at 4 °C overnight with antibodies against HA(1:1000, 11,867,431,001, Roche). After being washed with PBS 3 times for 5 min, the cells were incubated with secondary antibody (Rat, Alexa Fluor 488, Invitrogen, USA) for 1 h. Finally, the cells were visualized with a Leica TCS SP8 confocal laser scanning microscope (Leica TCS NT, Wetzlar, Germany) [9].

## Results

### Clinical data

**Fig. 1:**
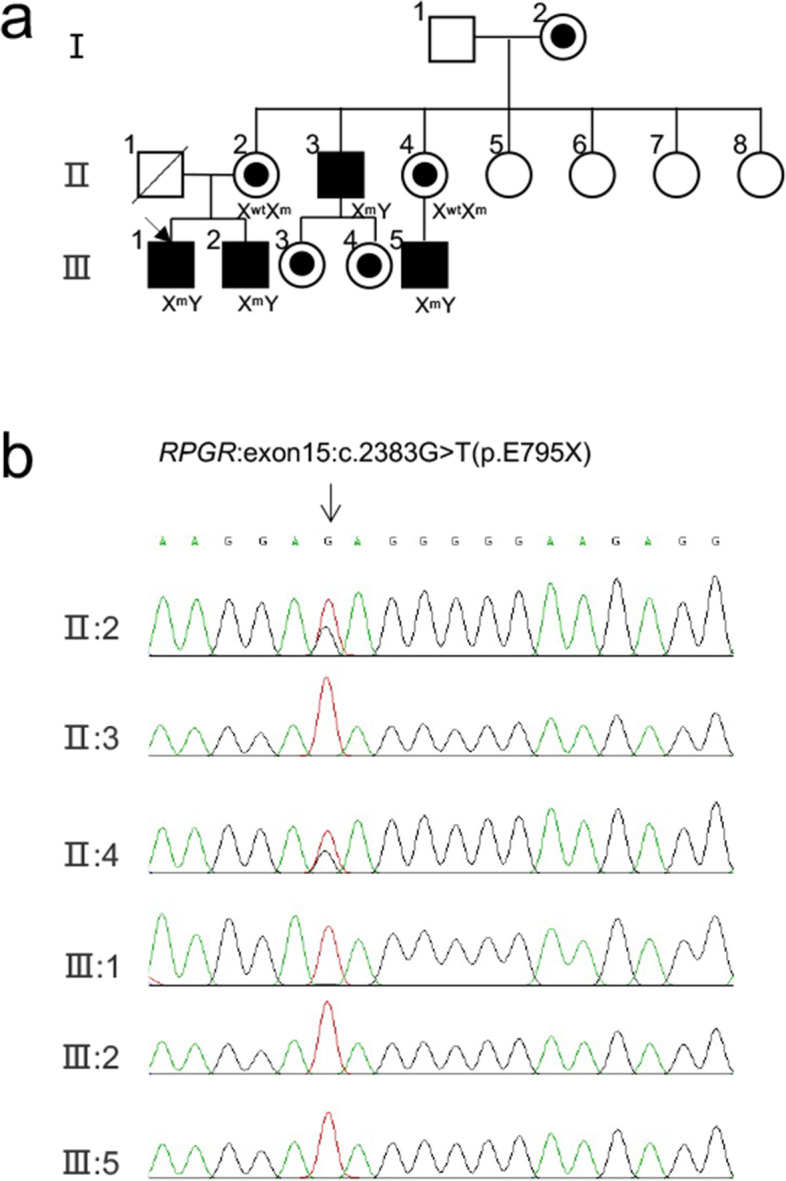
Family pedigree and DNA sequence of the proband (III:1). **a** Pedigree of the Chinese family with *RPGR*. Filled squares represent the affected individuals. Empty symbols represent the unaffected individuals, whereas empty symbols with black dot represent the carriers. All patients (II:3, III:1, III:2, and III:5) were identified as carrying hemizygous nonsense mutation (c.2383G > T, p.E795X) of *RPGR* in X chromosome. The members (II:2 and II:4) were identified carriers of the mutation. The members (I:2, III:3 and III:4) were inferred as carriers of the mutation. **b** DNA sequencing trace of the carriers (II:2 and II:4) and patients (II:3, III:1, III:2, and III:5) had the novel hemizygous nonsense mutation (c.2383G > T, p.E795X) of *RPGR*

**Fig. 2:**
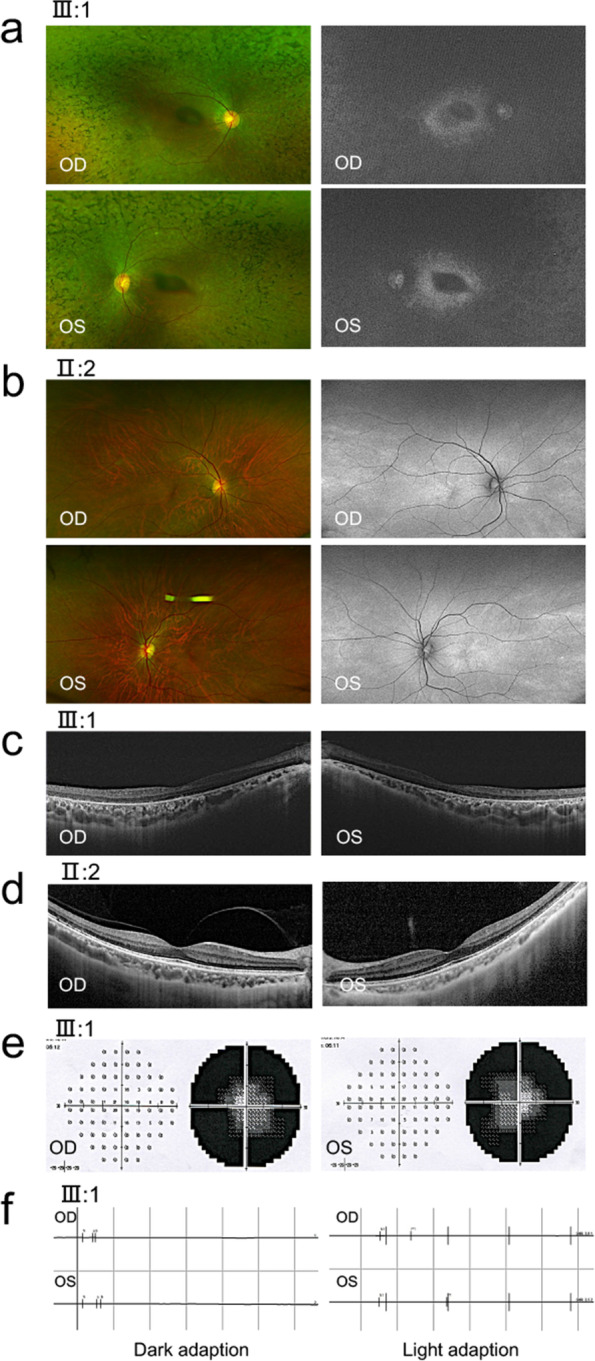
Clinical examinations of the proband and his mother. **a** The Optos widefield color fundus imaging of the proband (III:1, 32 years old) showed peripheral chorioretinal atrophy, macular dystrophy and mild RPE proliferation in the macular region in the proband. The autofluorescent imaging of the proband showed a hyperfluorescent lesion at the perifoveal and temporal quadrant areas and dot-like hypofluorescence around the lesion. **b** The Optos widefield color fundus imaging and the autofluorescent imaging of the carrier (II:2, 53 years old) were normal. **c**-**d** The OCT images of the proband (III:1) in the macular region showed outer retinal and choroidal atrophy, disrupted ellipsoid band and cavities in the choroid vessel and diffuse atrophy of Sattler layer. While the carrier (II:2) was normal. **e** The visual field examination suggested central and peripheral visual field defect of the proband (III:1). **f** ERG analysis demonstrated that the scotopic rod responses were undetectable, while the photopic responses were barely able to detect in the proband (III:1)

The pedigree is presented in Fig. 1a. All affected patients (II:3, III:1, III:2, and III:5) have suffered from typical symptoms for CORD, including early nyctalopia, progressive visual impairment, color vision defects and decreased sensitivity in the central visual field, followed by progressive loss in peripheral vision. The family members were examined by best-corrected visual acuity (BCVA), non-contact tonometer, widefield color fundus imaging, widefield fundus autofluorescence (FAF) and optical coherence tomography (OCT) scans. Besides, the affected patients were examined by visual field analysis and full-field electroretinogram (ERG). The ophthalmic diagnostic data were documented in Table 1 for four affected patients and two carriers, BCVA declined variously within all affected patients, while their intraocular pressure appeared normal. Bone spicule pigmentation, severe peripheral chorioretinal atrophy, macular dystrophy and RPE proliferation were noticed in the macular region of the proband (Fig. 2a). Moreover, by autofluorescence imaging, hypofluorescent lesions were found at the perifoveal, peripheral and posterior regions (Fig. 2a). It also revealed surrounding hyperautofluorescent rings (Fig. 2a). We further confirmed the existence of atrophy in the outer retinal layer, choroid, Sattler’s layer and disrupted ellipsoid zone and cavities in the choroidal vessels by OCT imaging (Fig. 2c). Nearly whole visual field has been lost for the proband, we could surmise the loss of central visual filed was followed by peripheral visual filed with his symptoms. (Fig. 2e). ERG analysis of the proband demonstrated that neither scotopic nor photopic response was barely detectable (Fig. 2f). While the carrier’s (II:2) fundus pictures and OCT were normal (Fig. 2b,d).

**Table 1:** The information and examinations of the family

Individual	Age	Gender	CORD symptoms	BCVA (OD/OS)	IOP (OD/OS mmHg)	Fundus	FAF	OCT
II-2	55	F	no	1.0/1.0	16/16	normal	normal	normal
II-3	50	M	early nyctalopia, progressive visual impairment, color vision defects, decreased sensitivity in the central visual field, followed by peripheral vision	Fc/30 cm/Fc/20 cm	12/15	bone spicule pigmentation	hypofluorescent lesion at the perifoveal region	atrophy in the outer retinal layer
II-4	47	F	no	0.8/0.6	17/17	normal	normal	normal
III-1	32	M	early nyctalopia, progressive visual impairment, color vision defects, decreased sensitivity in the central visual field, followed by peripheral vision	0.07/0.25	11/12	bone spicule pigmentation	hypofluorescent lesion at the perifoveal region	atrophy in the outer retinal layer
III-2	19	M	early nyctalopia, progressive visual impairment, color vision defects, decreased sensitivity in the central visual field, followed by peripheral vision	0.04/0.04	17/16	bone spicule pigmentation	hypofluorescent lesion at the perifoveal region	atrophy in the outer retinal layer
III-5	23	M	early nyctalopia, progressive visual impairment, color vision defects, decreased sensitivity in the central visual field, followed by peripheral vision	0.25/0.3	15/18	bone spicule pigmentation	hypofluorescent lesion at the perifoveal region	atrophy in the outer retinal layer

### Identification of RPGR mutation

**Fig. 3:**
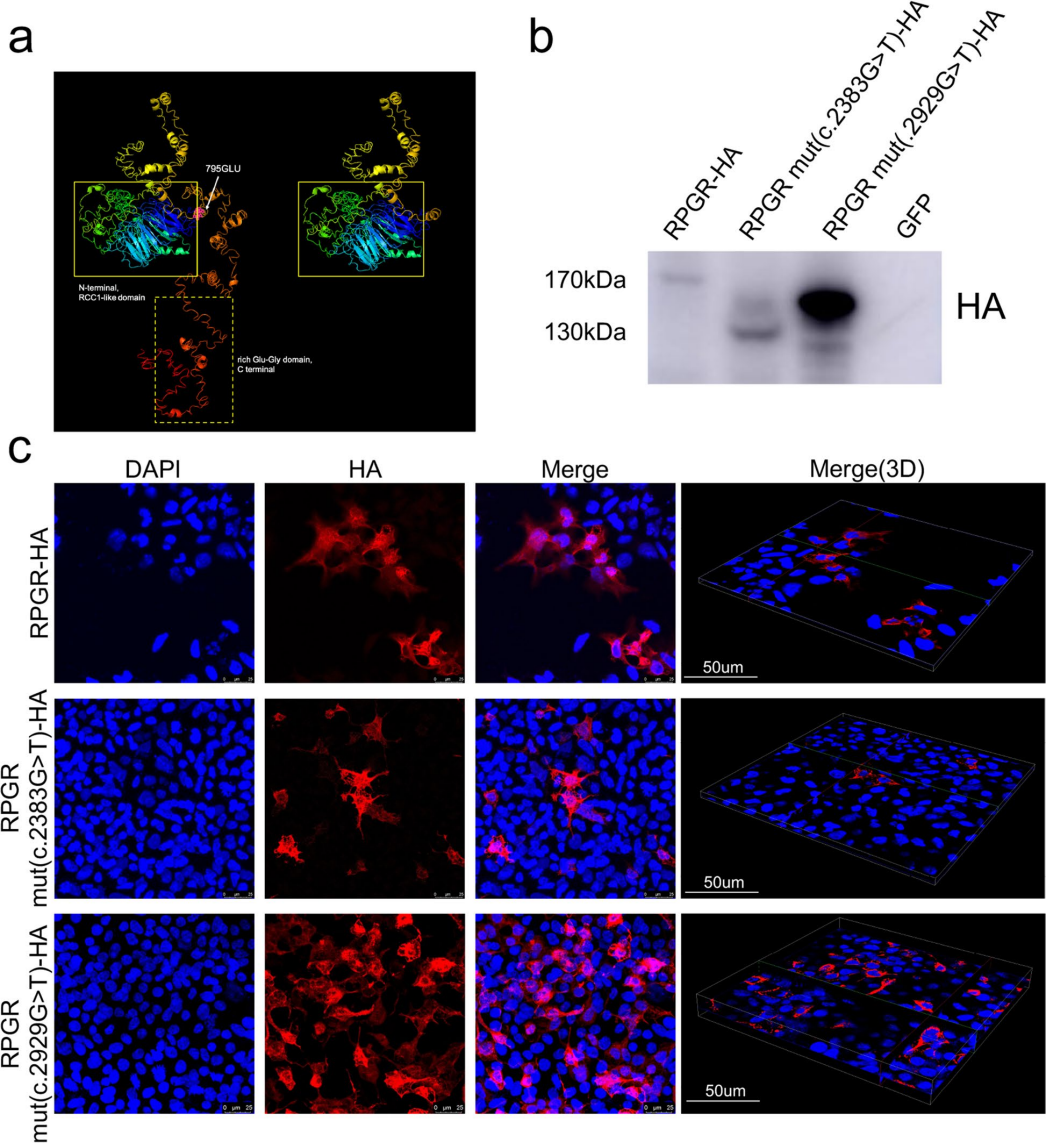
The c.2383G > T, p.E795X nonsense mutation in RPGR. **a** Structural comparison between wild type and mutant *RPGR* protein. Wild type (left) and mutant (right) *RPGR* protein models are shown. While the N-terminal RCC1-like domain is conserved (highlighted by yellow boxes), the hemizygous mutation induces the loss of a long C-terminal domain including a highly rich of the glutamic acid domain (dotted box), resulting in a much shorter protein. **b** Expression of RPGR-wt, RPGR-mut (c.2383G > T) and RPGR-mut (c.2929G > T) in transfected 293 T cells was assessed by Western blot. The grouping of blot was cropped from the same gel. The full-length blot was included in a supplementary information file. **c** Confocal images show both RPGR-wt, RPGR-mut (c.2383G > T) and RPGR-mut (c.2929G > T) appeared in the cytoplasm

In order to study the genetic basis for the affected patients, we collected peripheral blood samples from the proband for WES analysis. The average sequence coverage was over 100X and more than 98% of target bases were covered with at least 70X. A novel variation in *RPGR* gene (c.2383G > T) was identified as the potential disease-causing mutant, which results in a nonsense codon (p.E795X) and premature translational termination. The novel *RPGR* variant has not been reported in the human gene mutation database (HGMD). We further screened the genomic DNAs of the family by polymerase chain reactions followed by Sanger sequencing. As shown in Fig. 1b, the novel *RPGR* variant c.2383G > T was confirmed for the proband and the other affected family members as a hemizygous mutation. On the other hand, two unaffected members (II:2 and II:4) were hemizygous carriers of the mutation. The female members (I:2, III:3 and III:4) were asymptomatic, and they were inferred as hemizygous carriers according to Mendelian inheritance. Therefore, the novel *RPGR* variant c.2383G > T co-segregates with affected patients. The c.2383G > T mutation results in almost complete loss of *RPGR* exon ORF15 (p.E795X). We model the structure of *RPGR* with I-TASSER web server. As shown in Fig. 3a, full-length *RPGR* polypeptide is composed of RLLC-like, Glu-Gly-rich domains. The c.2383G > T, p.E795X mutation results in truncation of the Glu-Gly-rich and C-terminal domains. Based on these findings, we concluded that the *RPGR* variant c.2383G > T as identified in our study represents a novel disease-causing mutation.

### The c.2383G > T nonsense mutation results in truncated RPGR mutant

In order to indentified the function of the nonsense mutated protein, c.2383G > T mutated RPGR, c.2929 G > T mutanted RPGR (a previous reported known mutation [10]), and wild-type RPGR were sub-cloned into a mammalian expression vector (AAV-MCS) with an HA tag. The constructs were transiently transfected into 293 T cells and tested by Western blotting with cell lysates. As shown in Fig. 3b, the c.2383G > T mutation results in an about 40 kDa truncation of protein. Immunostaining of transfected cells with HA antibodies showed that there was no difference in the protein location in cells between RPGR-wt, RPGR-mut (c.2383G > T), and RPGR-mut (c.2929G > T). (Fig. 3c).

## Discussion

In this study, we report a Han-Chinese family diagnosed with XLCORD. Complete clinical diagnostic data are presented. The further genetic analysis uncovered a novel nonsense variant (c.2383G > T, p.E795X) in *RPGR* gene that co-segregates with the affected male patients as hemizygotes and unaffected females as heterozygous carriers. Thus, the variant c.2383G > T in *RPGR* gene represents a novel disease-causing mutation leading to X-linked CORD.

**Fig. 4:**
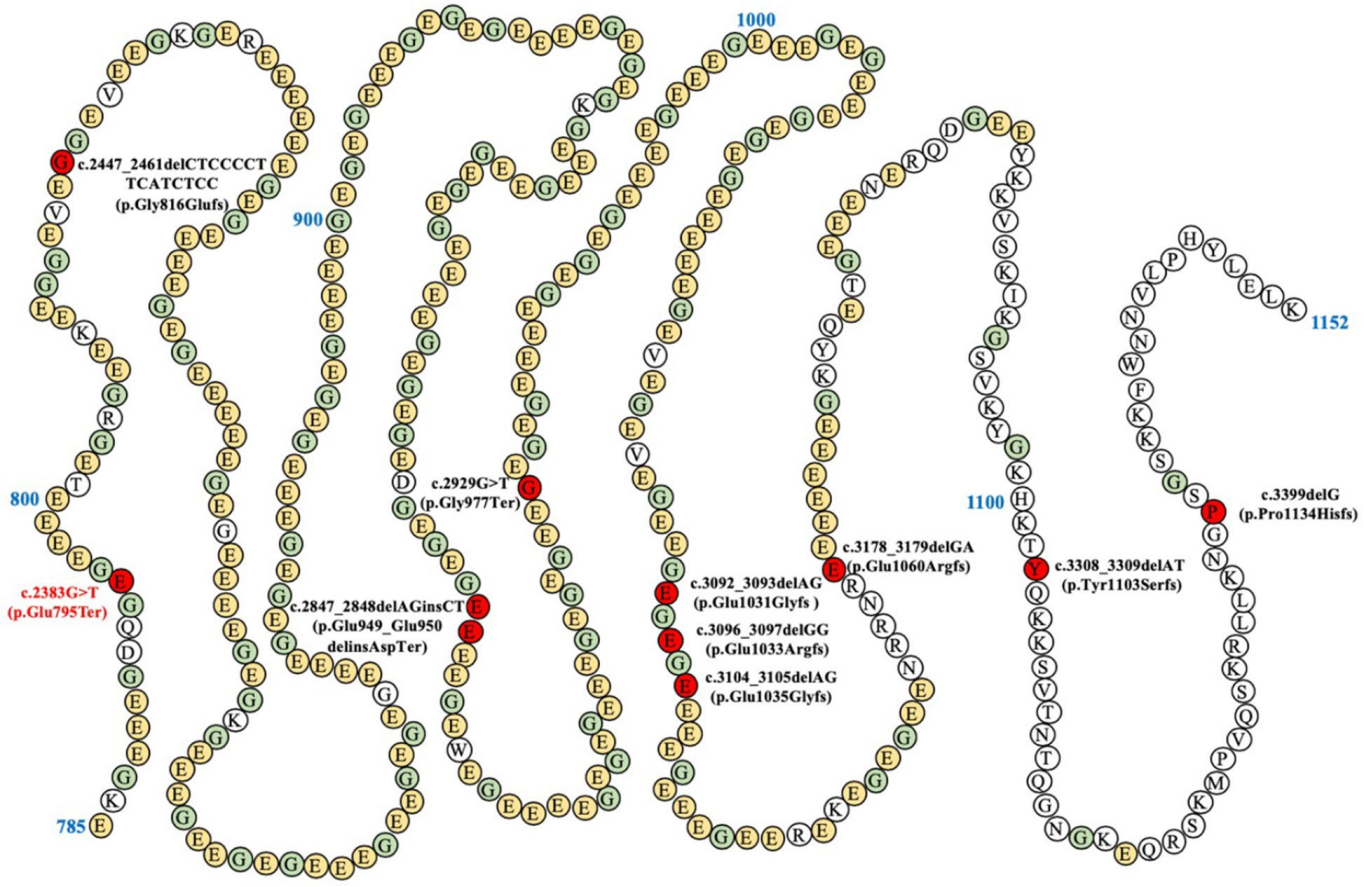
A representation of *RPGR* pathogenic mutations in the exon ORF15 causing CORD reported thus far. The animo acid mutations were marked in red. The novel mutation was presented in bold&red font

*RPGR* is a major mutagenic locus for X-linked CORD. Pathogenic mutations that caused CORD (reported in the literature and sorted by mRNA Sequence from ClinVar) are preferentially sequestered at the 3′ end of the ORF15 region in *RPGR* [10], as illustrated in Fig. 4 and Table 2. *RPGR* is expressed in the transition zone and basal bodies of photoreceptor cilia [11]. As a regulator of protein trafficking along the photoreceptor cilium, *RPGR* is involved in maintaining the structure and function of mature cilia. Defects in *RPGR* result in a minimal but incremental lesion, which finally results in retinal degeneration [12]. The nonsense mutation (c.2383G > T, p.E795X) as identified in this study is located in exon ORF15, the so-called ‘mutational hotspot’ where highly repetitive purine-rich sequences are recognized as a mutagenesis-prone region [13]. The c.2383G > T, p.E795X mutation results in truncation of *RPGR* polypeptide without the Glu-Gly-rich domain at the C-terminus. The N-terminal domain (RLD), which features a beta-propeller structure composed of seven blade-shaped beta-sheets, is preserved in the truncated mutant (Fig. 3a). The RLD domain plays a role in *RPGR* localization to cilia by binding to *RPGR*IP1 and *RPGR*IP1L [14]. In addition, the RLD domain mediates complexation of *RPGR* with SMC1/3, PDEδ, and Rab8 that are critical to cilia functions [12, 15]. Mutations in the C-terminal Glu-Gly-rich domain affect *RPGR* glutamylation, although its functional outcome is yet to be identified [16]. Moreover, the C-terminus of *RPGR* interacts with whirlin, mutations of which result in Usher syndrome, and nucleophosmin, which functions in cell division [17, 18]. Given the complexity of a *RPGR* interactive network, the pathogenic mechanism underlying *RPGR* mutations remains to be clarified.

**Table 2:** Summary of pathogenic mutations caused CORD reported thus far

Nucleotide and amino acid changes	Patient age	Patient population	Literature PMID
NM_001034853.1(RPGR): c.2447_2461delCTCCCCTTCATCTCC(p.Gly816Glufs)	40/not provided	2	11,875,055
NM_001034853.1(RPGR):c.2847_2848delAGinsCT (p.Glu949_Glu950delinsAspTer)	37,40,48,51,74	5	15,914,600
NM_001034853.1(RPGR):c.2929G > T(p.Gly977Ter)	22,44,50,70,73	8	15,914,600
NM_001034853.1(RPGR):c.3092_3093delAG (p.Glu1031Glyfs)	not provided	not provided	11,857,109
NM_001034853.1(RPGR):c.3096_3097delGG (p.Glu1033Argfs)	47/not provided	2	32,047,640
	47	not provided	11,857,109
	50,74	2	11,875,055
NM_001034853.1(RPGR):c.3104_3105delAG (p.Glu1035Glyfs)	11	2	32,047,640
NM_001034853.1(RPGR):c.3178_3179delGA (p.Glu1060Argfs)	50	1	32,047,640
NM_001034853.1(RPGR):c.3308_3309delAT (p.Tyr1103Serfs)	74	1	32,047,640
NM_001034853.1(RPGR):c.3399delG(p.Pro1134Hisfs)	57	2	32,047,640

It was not clear why some *RPGR* mutations cause RP while others cause CORD until now. While it seems reasonable to speculate that mutations in the exon ORF15 are more often found in CORD cases, whereas mutations in the exon 1–14 are more often found in retinitis pigmentosa (RP). In contrast to typical CORD, RP results from the primary loss in rod photoreceptors and later followed by the secondary loss in cone photoreceptors. Cross-sectional analyses suggested that patients of RP with mutations in exons 1 to 14 were more likely to have the severe symptoms than the patients with ORF15 mutations [19, 20]. Consistent with this, our study demonstrated that both female carriers showed normal phenotype with mutation in ORF15 (Fig. 2b,d). In contrast, previous report from Yang LP et al. [21] demonstrated that the heterozygous females who suffered the mutations in exon 8 implicated high myopia accompanied by severe retinal and visual function abnormalities in one eye. For their nonsense mutation led to the disruption of RCC1-like domain, the functional loss of RPGR with other interaction partners was the most probable reason. In addition, we demonstrated our nonsense mutation created the truncated protein, not nonsense-mediated decay, and the shortened protein didn’t contain the Glu-Gly-rich domain (Fig. 3).

The exact mechanism of the ORF15 played a significant role in this pedigree remains unclear, which needs to be further investigated. Last but not least, clinical transformation research on treatment methods also has a long way to go.

## Conclusions

In summary, our findings have identified a novel point mutation in *RPGR*, c.2383G > T, p.E795X on chromosome X that contributes to X-linked CORD and further broadens the spectrum of RPGR mutations.

## Supplementary Information

**Additional file 1.**

## Data Availability

The data of whole-exome sequencing of the proband in this study was deposited in Dryad (DOI (doi:10.5061/dryad.5qfttdz5d)).

## Abbreviations

CORD: Cone-rod dystrophy
XLCORD: X-linked cone-rod dystrophy
IRDs: Inherited retinal dystrophies
ERG: Electroretinogram
RPGR: Retinitis pigmentosa GTPase regulator
BCVA: Best-corrected visual acuity
FAF: Fundus autofluorescence
OCT: Optical coherence tomography
WES: Whole-exome sequencing
HGMD: Human gene mutation database
RPE: Retinal pigment epithelium
RP: Retinitis pigmentosa
