# Supplementary figures and images for: Identification of a novel RPGR mutation associated with X-linked cone-rod dystrophy in a Chinese family
Source: BMC Ophthalmol. 2021 Nov 20;21:401. doi: 10.1186/s12886-021-02166-0 (PMC8605601; doi:10.1186/s12886-021-02166-0)

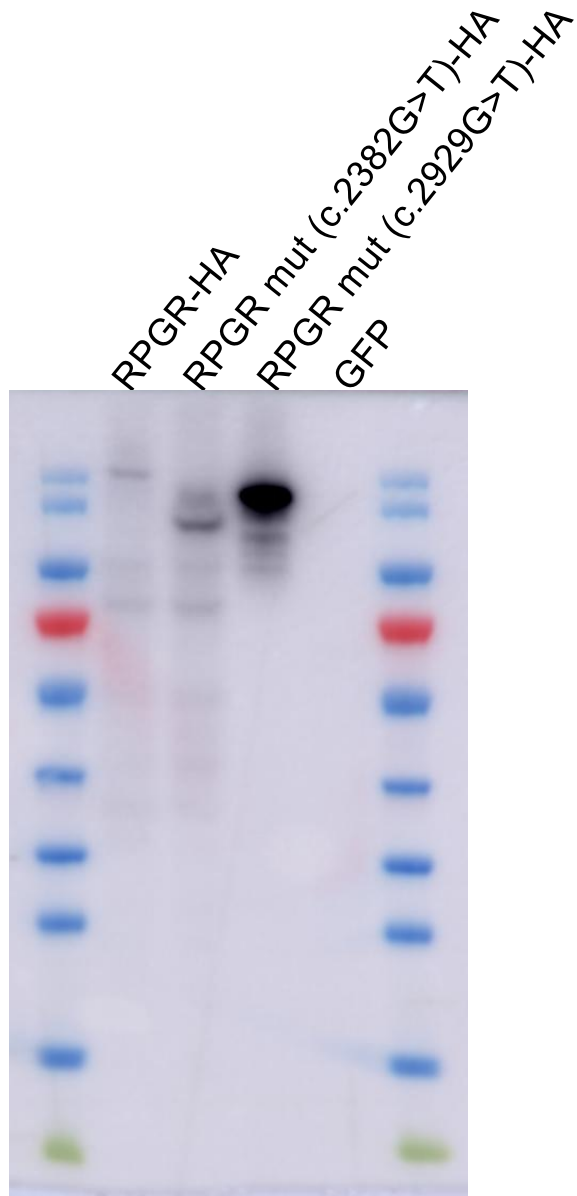

The full-length blot of Fig. 3b.

Supplement: Supplementary file 1 — Additional file 1. [file 12886_2021_2166_MOESM1_ESM.pdf]
